# Selective Impact of MTMS-Based Xerogel Morphology on Boosted Proliferation and Enhanced Naphthoquinone Production in Cultures of *Rindera graeca* Transgenic Roots

**DOI:** 10.3390/ijms232213669

**Published:** 2022-11-08

**Authors:** Kamil Wierzchowski, Bartosz Nowak, Mateusz Kawka, Patryk Więckowicz, Katarzyna Dąbkowska-Susfał, Agnieszka Pietrosiuk, Katarzyna Sykłowska-Baranek, Maciej Pilarek

**Affiliations:** 1 Faculty of Chemical and Process Engineering, Warsaw University of Technology, Waryńskiego 1, 00-645 Warsaw, Poland; 2 Department of Biology and Pharmacognosy, Faculty of Pharmacy, Medical University of Warsaw, Banacha 1, 02-097 Warsaw, Poland

**Keywords:** xerogel, MTMS, transgenic roots, hairy roots, naphthoquinones, in situ extraction

## Abstract

In situ extraction is a method for separating plant secondary metabolites from in vitro systems of plant biomass cultures. The study aimed to investigate the MTMS-based xerogels morphology effect on the growth kinetics and deoxyshikonin productivity in xerogel-supported in vitro culture systems of *Rindera graeca* hairy root. Cultures were supplemented with three types of xerogel, i.e., mesoporous gel, microporous gel, and agglomerated precipitate, in the disintegrated or monolithic form. Structure, oil sorption capacity, and SEM analyses for xerogel-based additives were performed. Application of monolithic macroporous xerogel resulted in the highest biomass proliferation, i.e., 5.11-fold fresh biomass increase after four weeks of the screening culture. The highest deoxyshikonin production (i.e., 105.03 µg) was noted when hairy roots were maintained with particles of disintegrated mesoporous xerogel. The detailed kinetics investigations (6-week culture) revealed the highest growth of hairy root biomass and secondary metabolite production, equaling 9.46-fold fresh weight biomass and 204.08 µg deoxyshikonin, respectively. MTMS-based xerogels have been recognized as selective biocompatible scaffolds for boosting the proliferation of transgenic roots or for productivity enhancement of naphthoquinones without detrimental effects on biomass growth, and their successful applicability in in situ removal of secondary plant metabolites has been experimentally confirmed.

## 1. Introduction

Pathways of secondary metabolism in plant cells provide a prolific array of bioactive compounds responsible for the specialized adaptation of the plant to live in a given ecosystem niche. The biological activity of plant secondary metabolites implicates the vast potential of their practical and prospective applications as active pharmaceutical or cosmetic ingredients, food additives, or agricultural biochemicals [1,2,3]. Due to low concentrations of secondary plant metabolites typically occurring in biomass harvested from available natural resources, the commercial feasibility of their industrial manufacturing is often limited [4]. However, modernly, the efficiency of pharmaceutically relevant metabolites can be intensified by biotechnological or bioengineering methods [5].

Many published review articles and a wide range of scientific reports notify that the solution for most of the obstacles related to plant biomass harvesting for secondary metabolites production comes with in vitro bioprocessing of hairy roots [6,7,8,9]. Due to stable genetic alterations induced by *Rhizobium rhizogenes*, hairy root features intensified growth in a culture media free of plant growth regulators, with the propensity for lateral branching and enhanced biosynthesis of secondary metabolites specific for root tissue differentiation [10]. Such a beneficial phenotype of transgenic hairy roots fits well for industrial applications of in vitro plant biomass cultures, making it possible to conduct validated scaled-up bioprocesses in bioreactors (e.g., disposable aero- or hydroponic systems) and providing the tool to overcome ecological or economic limitations [11,12]. Moreover, the unique but end-user-defined parameters of a strictly controlling bioprocess environment allow for various culture system modifications. Among reported techniques, much attention is dedicated to in situ separation of bioproducts, defined as a process of directed metabolite accumulation within an additional phase (i.e., liquid extractant or solid-state adsorbent) added into the culture medium [13,14,15]. The desired selectivity of in situ applied extractant/adsorbent toward a specific metabolite is favorable for production efficiency because it enables enhanced secretion of bioproducts through its selective separation from the culture medium, resulting in the cancellation of separated metabolite biosynthesis inhibition, which finally resulted in increased yield of its production [16].

The application of solid materials for hairy root immobilization has been studied only in a few papers. Srivastava noticed the first application of solid material for cultures of hairy root in 2012 for cultures of *Azadirachta indica* root biomass on the polyurethane foam (PUF) [17]. In addition, in 2017, Thakore applied PUF for the *Catharanthus roseus* hairy root cultures [18]. In both studies, the root biomass growth on PUF scaffolds was inhibited, but the application of PUF increased metabolite production. In the case of the other materials, polypropylene (PP) was used for hairy root biomass immobilization for supporting *Azadirachta indica* root biomass [19] and *Plumbago rosea* hairy roots [20]. In this study, the PP scaffolds were inert for hairy roots, but the production of metabolites was not satisfactory.

Considering both problems mentioned above in root biomass immobilization, novel, non-toxic biomaterials, which induce the biosynthesis of metabolites, are strongly needed. In our previous proof-of-concept study, methyltrimetoxysilane (MTMS)-based gels, with their highly porous structure, hydrophobicity, and biocompatibility, fulfilled conceptual requirements. Previously published research aimed to check the application of MTMS-based gel constructs as support for *R. graeca* hairy root immobilization and in situ extraction of naphthoquinones. The studied material exhibited superiority over other investigated materials for in situ product separation of naphthoquinone compounds from in vitro culture of *Rindera graeca* hairy roots, i.e., PUF and PP [21].

**Figure 1:**
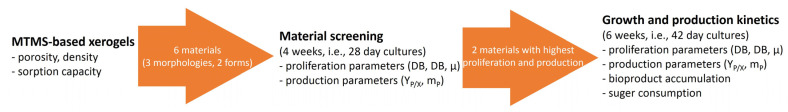
Schematic presentation of investigation concept.

The scope of the presented study has been presented in Figure 1. The first stage (i.e., screening stage, 4-week cultures) of the study was focused on recognizing the selective effects of MTMS-based xerogel morphology as a boosting agent for proliferation and enhanced production of deoxyshikonin in cultures of *R. graeca* hairy roots. In the second stage of the study (i.e., optimization stage, 6-week cultures), hairy root proliferation and deoxyshikonin production in culture systems supported with the most promising MTMS-based xerogels were comprehensively analyzed. In our opinion, the concept presented in this study is a breakthrough experimental technique for hairy root biomass cultivation and for the increment of plant secondary metabolites biosynthesis, which broadly interests the plant biologists and scientists involved in receiving biologically active compounds.

## 2. Results

### 2.1. Xerogels Characterization

**Figure 2:**
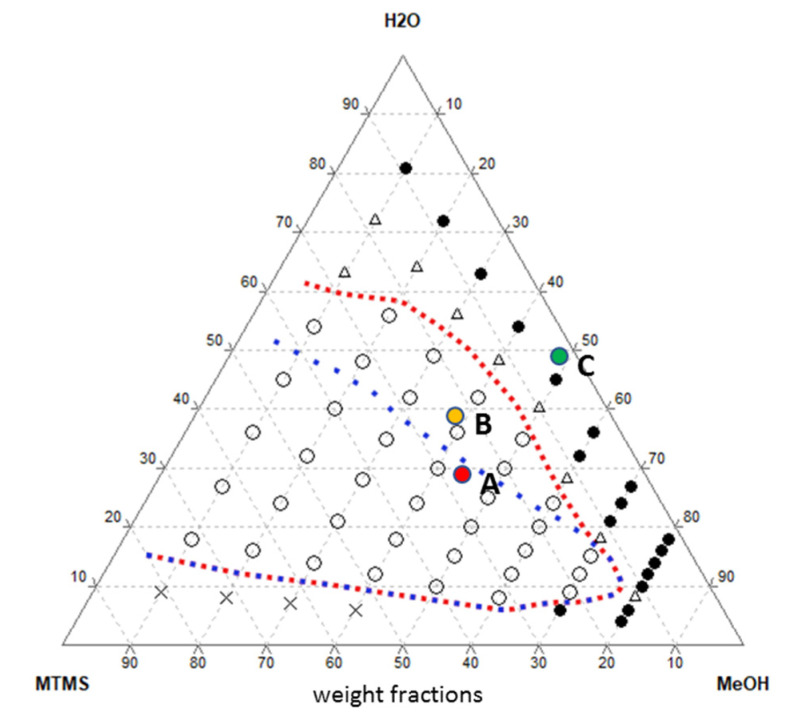
Sol–gel synthesis of MTMS-based gels described on the ternary system (Gibbs triangle), with synthesis composition for investigated samples A–C. Red line—binode, blue line—spinode, ✕—silica resin, ○—monoliths, Δ—macroscopic phase separation, ●—precipitate.

MTMS-based gels varying in proportion of reagents used for their synthesis (as listed in Section 4) were chosen to investigate the impact of structural differences of xerogel-based materials on the efficiency of *R. graeca* hairy root cultures. Utilizing the Gibbs triangle (shown in Figure 2), one can track how the composition of the reactive mixture influences the final gel morphology [22,23]. The binode line (red dotted line) stands for the synthesis range of monolithic gels, which corresponds to stable and metastable regions. A and B synthesis proportions are inside this region, although they are separated by the spinode (marked as a blue dotted line). The spinodal curve represents the boundary between metastable and unstable regions [24,25,26], which marks the transition between the phase separation mechanisms—nucleation and growth (between spinode and binode) and spinodal decomposition (inside the spinode) [27]. Nucleation and the growth mechanism lead to a particle aggregate type of structure morphology, while through the spinodal decomposition, co-continuous, nanoporous, or isolated pores structure can be obtained [28]. The synthesis proportions of sample C are outside the binodal region; hence, a gel monolith can not be formed. Instead, synthesis of the precipitate or its agglomerates occurs in these conditions.

**Figure 3:**
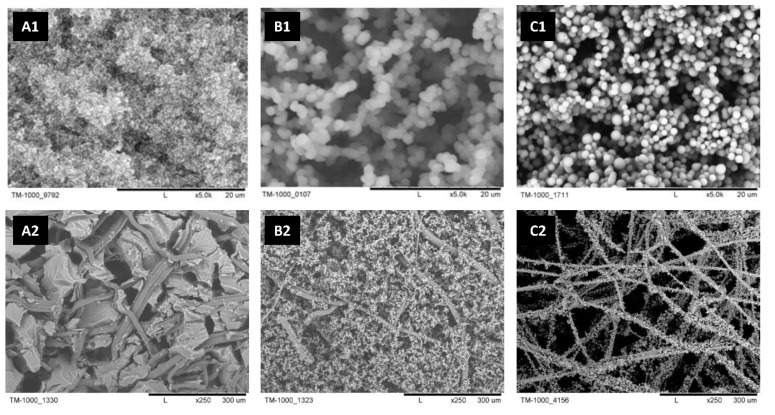
The SEM micrographs documenting the morphology of experimentally investigated MTMS-based materials: (**A1**–**C1**) gels, (**A2**–**C2**) PP-reinforced MTMS-based constructs.

The morphological differences between samples are shown in Figure 3. The SEM micrographs of A1 and B1 samples revealed the mesoporous (A1) and macroporous (B1) structures of gels applied in disintegrated form. The morphology of the presented gels differed because of the various volume fractions of catalyst aqueous solution used in the gelating mixture [29], where methanol is a solvent for organosilica precursor, and water acts as a non-solvent. Thus, water can be considered a porogenic agent during microscopic phase separation during gel condensation. Therefore, a higher volume of aqueous solution applied in the synthesis of sample B1 resulted in the formation of larger secondary particles and larger pore size characterizing sample B1 than in mesoporous sample A1. The concentration of MTMS in the C1 sample was too low to form a stable siloxane network in the whole volume of condensing mixture (please compare to [22,29]). Instead of monolithic gels, the C1 consists of agglomerated precipitate of secondary particles, much more considerable in diameter than sample A1.

While deposited on PP fibers (A2–C2), MTMS-based xerogel maintains its micro-scale structures. In the macroscale (the bottom row in Figure 3), samples show differences in the arrangement of the fibers. The A2 sample exhibits cracked gel that fills the PP-based fabric. On the other hand, the B2 sample has a continuous structure of xerogel located between and around PP fibers, forming macropores. The C2 resembled a visible mat structure, with secondary particles forming a porous layer directly on the surface of the fibers made of PP.

Differences in the morphology of the A2–C2 sample can be explained via the gel behavior during the drying procedure. The smaller the gel pores, the greater the capillary pressure, and hence, the more difficult it is to remove the solvent from the material volume [30,31]. The use of the ambient pressure drying (APD) method results in gel volume shrinkage caused by mentioned capillary forces; hence, the highest value of gel volume shrinkage, reaching 58%, was identified for the synthesis of MTMS-based gel A2 (see Table 1). Lower volume shrinkage was observed for the B2 sample—reaching approximately 28.5%. Due to macroscopic phase separation, gel in the C2 sample did not form a monolith but condensed into loose secondary particle aggregates. Due to volume shrinkage during the APD method and friction between fibers and gel, the structure leaves empty spaces between fibers, shrinking to their surface. Therefore, the final structure of material C2 takes a form of a fiber-surrounding layer. All the effects observed during the drying MTMS-based materials studied in this work are consistent with the PP/MTMS–gel connection phenomena described in detail by Nowak et al. [32].

The total condensing solution volume applied in synthesizing A2–C2 materials was constant, equaling 150 mL for 15 cm diameter fabric. As the concentration of MTMS decreased, so did the mass of the gel deposited on the PP non-woven fabric and the packing density of the final construct (see Table 1). PP/MTMS construct A2 exhibited the highest MTMS–gel to PP fibers weight ratio and packing density, followed by samples B2 and C2. The packing density of the native PP matte is listed as a reference.

**Figure 4:**
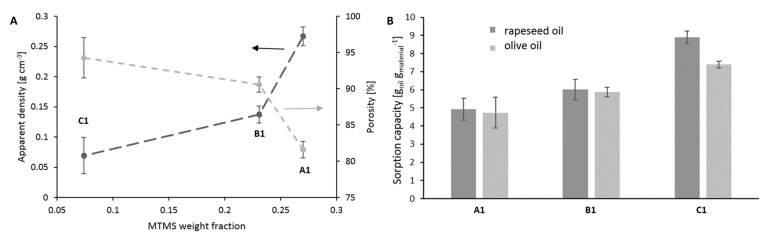
Used MTMS-based materials’ (synthesis A1–C1, as shown in Figure 2) apparent density (black line) and porosity (grey line) in the function of MTMS weight fraction (**A**), rapeseed oil, and olive oil sorption capacity of investigated gels (**B**).

Applied synthesis methodology and volume shrinkage resulting in the APD method affected the gel morphology and its sorption capacity. In Figure 4A, the dry gel’s apparent density and porosity are presented as a function of MTMS weight fraction. The decrease in the MTMS weight fraction comes along with less solidity in obtained wet gels, which leads to a lower apparent density of dried materials [33]. The highest concentration of MTMS and the highest volume shrinkage (see Table 1) caused by a small pore diameter for gel A1 leads to a high-value apparent density and porosity equal to approximately 82.5%. As the pore size of gel B1 is in the range of small macropores, volume shrinkage is significantly lower, and xerogel porosity reaches above 90%. The lowest apparent density, the highest porosity value—94.7%, was measured for gel C1. In the case of sample C1, apparent density can be understood as bulk density.

The MTMS–gel porosity is correlated with the oil sorption capacity [34], as presented in Figure 4B. The gel A1 exhibited the lowest sorption capacity for both investigated lipid fractions, reaching approximately 4.5–5 g g^−1^. Next was gel B1, followed by gel C1—about 6 and 7–9 g g^−1^, respectively. All measured sorption capacities exceed the root production capacity, as will be shown later.

### 2.2. Physiological Effects Resulting from In Vitro Supporting R. graeca Hairy Roots with Various MTMS-Based Materials (The 28-Day Screening Cultures)

**Figure 5:**
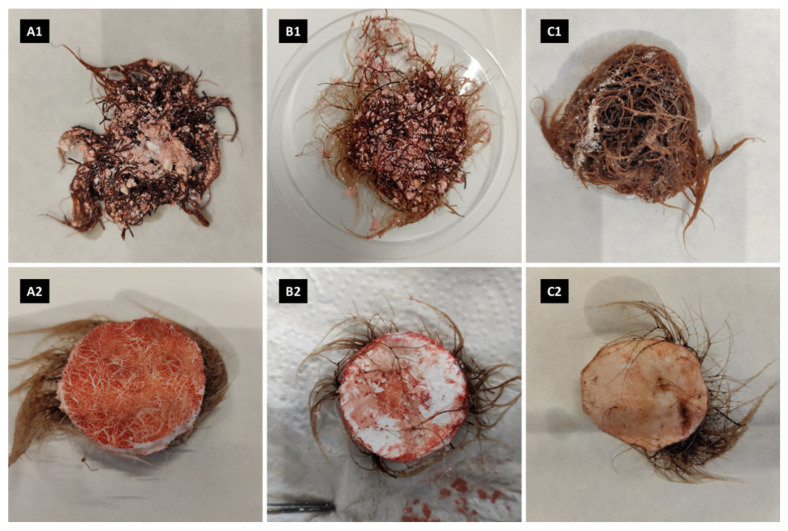
Morphology of *R. graeca* hairy roots in vitro cultured for 28 days with investigated MTMS-based materials: disintegrated mesoporous xerogel (**A1**), disintegrated macroporous xerogel (**B1**), an aggregated precipitate of MTMS (**C1**), PP-reinforced mesoporous xerogel (**A2**), PP-reinforced microporous xerogel (**B2**), PP fibers with a deposited precipitate of MTMS (**C2**).

Images reporting the morphology of *R. graeca* hairy roots in vitro cultured for 28 days with six investigated MTMS-based materials are presented in Figure 5. In the case of materials applied in disintegrated forms (Figure 5(A1–C1)), the amount of the residual xerogel in the biomass–gel conglomerate was unequal, with the highest amount of adhering MTMS-based material observed for the culture system supplemented with xerogel A1. For xerogel B1, the biomass contained fewer adhered gel particles, but the hairy roots maintained with an aggregated precipitate of MTMS (i.e., material C1) exhibited the lowest amount of biomass. The hairy roots adhered to the constructs applied in monolithic forms of MTMS-based gels reinforced with PP fibers (Figure 5(A2,B2,C2)). Due to the robust adherence of gels in disintegrated and integrated forms to the surface of hairy roots, biomass separation from MTMS-based materials without its destruction was impossible. The separation of MTMS-based elements from hairy root biomass caused the peeling of the top layer of the monolithic gel-based structure in all investigated cases. In addition, after 28 days of culture, all MTMS-based materials, originally white-colored, were tinted with various shades of red. Deoxyshikonin is a deep red dye; thus, the basic qualitative analysis of deoxyshikonin production, and its sorption by studied materials, may be compared to differences in the coloring of xerogels. Particles of disintegrated gels A1 and B1 were colored almost equally, unlike almost undyed particles of aggregated precipitate of MTMS C1. The most intense, red-dyed material was noticed for construct A2, material B2 was less severely colored, and for construct C2, the red color was only slightly visible.

**Figure 6:**
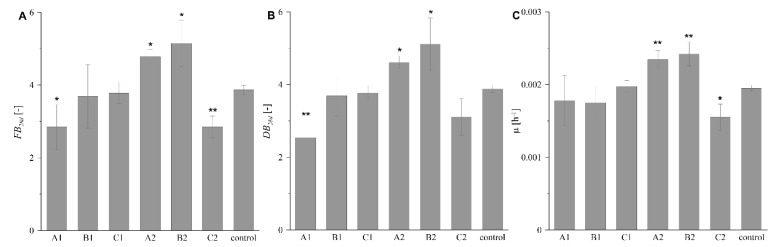
Values of FB_28d_ (**A**), DB_28d_ (**B**), and µ (**C**) quantitatively identify the proliferation of *R. graeca* hairy roots in culture systems varying in the form of supporting MTMS-based gel and in the control culture without any MTMS-based material. Following materials were investigated: (A1) disintegrated mesoporous xerogel, (B1) disintegrated macroporous xerogel, (C1) aggregated precipitate of MTMS, (A2) PP-reinforced mesoporous xerogel, (B2) PP-reinforced microporous xerogel, (C2) PP fibers with a deposited precipitate of MTMS. The asterisks mark the culture systems at which the determined values of the parameters were statistically different from the values identifying the reference culture with *p* < 0.05 (*) and *p* < 0.01 (**) according to the ANOVA test.

The quantitative data identifying the proliferation of *R. graeca* transgenic roots maintained with the six investigated MTMS-based gels and in the reference system without any scaffold, described as values of FB_28d_, DB_28d_, and µ, are presented in Figure 6. The highest values of the three proliferation parameters (i.e., FB_28d_, DB_28d_, µ) were observed for the culture system supported with gel B2. Slightly lower values of those parameters gave culture supported with gel A2. The values of proliferation parameters reported for systems supplemented with PP-reinforced gels B1 and C1 were similar to values obtained for the reference culture. The values of FB_28d_, DB_28d,_ and µ for the system supported with gel A1 were noticeably lower than for other cultures supported with disintegrated gels. However, the lowest values of proliferation parameters were obtained in the culture system supported with gel C2.

**Figure 7:**
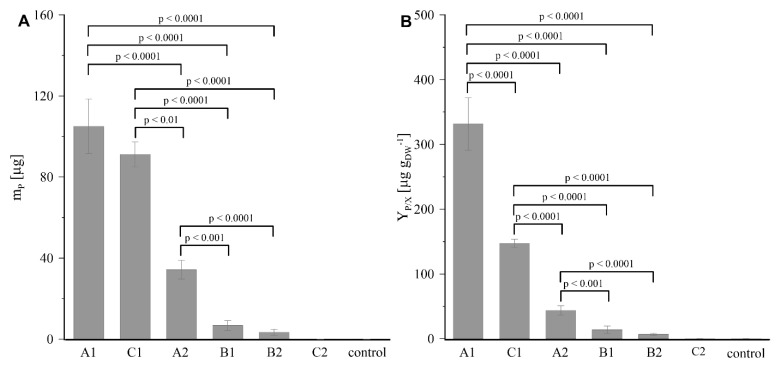
Values of m_P_ (**A**) and Y_P/X_ (**B**) obtained for cultures of *R. graeca* hairy roots supported with various MTMS-based materials and for the control culture: (A1) disintegrated mesoporous xerogel, (B1) disintegrated macroporous xerogel, (C1) aggregated precipitate of MTMS, (A2) PP-reinforced mesoporous xerogel, (B2) PP-reinforced microporous xerogel, (C2) PP fibers with a deposited precipitate of MTMS. The *p* values over brackets were determined according to the ANOVA test.

Values of m_P_ and Y_P/X_ identifying the production of deoxyshikonin by *R. graeca* transgenic roots independently cultured in six systems supported with MTMS-based materials and in the control culture are compared in Figure 7. The highest value of m_P_ was reported for the culture containing gel A1. The almost 1.2 times lower value of m_P_ was noticed for the culture supported with gel C1. The lowest m_P_ value for cultures supported with disintegrated forms of gels was observed for the system containing gel B1. For gels reinforced with PP fibers, the highest value of m_P_ exhibited the culture containing construct A2, which was three times lower than the value obtained in the system supported with material C1. A relatively meagre value of m_P_ (i.e., about 3 µg) was noted for the culture system with gel B2. In the PP-reinforced gel C2 system and the control culture, the m_P_ level did not pass the detection shoulder of the applied analytical method due to the lack of deoxyshikonin in both of these cultures. Moreover, the highest value of Y_P/X_ was observed for the culture system containing gel A1, and its value was over two times higher than the value characterizing culture supported with gel C1. The levels of Y_P/X_ calculated for culture systems supported with other MTMS-based materials were in line with the dependences of m_P_ values related to the applied form of MTMS-based gels described above.

Based on results presented in Figure 6 and Figure 7, the xerogels A1 and B2, the most efficiently stimulating *R. graeca* hairy roots for enhanced deoxyshikonin production and boosted biomass proliferation, respectively, were selected for further detailed study of their influence on biomass cultured in vitro. The cognitive reasons for xerogels A1 and B2 selection were the highest values of Y_P/X_ noted for the cultures supported with xerogel A1 and the highest values of FB_28d_, DB_28d_ and µ reported for cultures supported with xerogel B2.

### 2.3. Detailed Growth Kinetics and Deoxyshikonin Production in R. graeca Hairy Roots Cultures Supported with MTMS-Based Materials Providing Maximized Production of Biomass and Bioproduct

**Figure 8:**
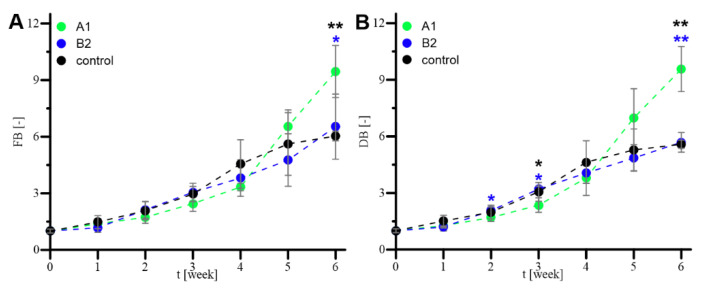
The values of FB (**A**) and DB (**B**) obtained for samples harvested daily from the prolonged 42-day (i.e., 6-week) cultures of *R. graeca* hairy roots supported with gels A1 or B2 and for the control culture. The asterisks mark the cultures at which the determined values of parameters were statistically different from the values characterizing the reference culture. The color of the asterisks is consistent with the color of the markers identifying the experimental data. The number of asterisks is related to the value of *p* (*p* < 0.05 (*) and *p* < 0.01 (**)) determined according to the ANOVA test.

The values of FB and DB obtained for samples harvested daily from the prolonged 42-day (i.e., 6-week) cultures of *R. graeca* hairy roots supported with gels A1 or B2 and for the control culture are presented in Figure 8.

For the first three weeks, the values of FB identifying all three compared culture systems, i.e., cultures supported with gels A1 and B2 and the control culture, were similar (Figure 8A). In the 4th week, the FB value noted for the control culture was slightly higher than the data for cultures supported with gels. Next, for the 5th and 6th weeks, the highest value of FB gave culture supported with xerogel A1, and the values of FB for the control culture and culture with gel B2 were almost equal. The value of FB noted for the 6th week of culture with gel A1 was over 35% higher than the value of the equivalent parameter reported for other cultures. Moreover, in the 6th week, the FB value for the control culture was the lowest among compared cultures, and it was nearly the FB value in the 5th week. Such observed effects may indicate the stationary phase of biomass growth in the control culture but continuing intensive hairy roots proliferation in the culture supported with gel A1. In the case of noted DB values, for the first four weeks, the DB values were similar (Figure 8B) regardless of the culture system, which was in accordance with the course of FB presented in Figure 8A. Next, in the 5th and 6th weeks, the DB values were significantly higher for the culture system supported with xerogel A1 than those noted for the control culture and the culture with xerogel B2.

**Figure 9:**
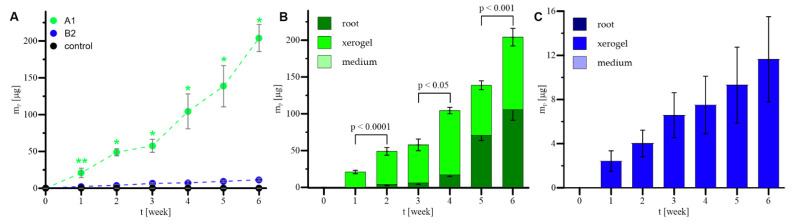
Values of m_P_ obtained for the prolonged 42-day (i.e., 6-week) cultures of *R. graeca* hairy roots supported with xerogels A1 and B2 xerogels, and the control culture: general comparison of the culture systems (**A**), detailed presentation of summarized production of deoxyshikonin including bioproduct accumulated in root biomass, xerogel and culture medium taken from systems supported with xerogels A1 (**B**) and B2 (**C**). The green asterisks mark the culture with gel A1 at which the determined values of studied parameters were statistically different from those for the B2 culture system. The number of asterisks identifying the value of *p* (*p* < 0.001 (**) and *p* < 0.0001 (*)), as well as the p values over brackets, were determined according to the ANOVA test.

The general m_P_ values obtained for the prolonged 42-day (i.e., 6-week) cultures of *R. graeca* hairy roots supported with xerogels A1 and B2 and the control culture are shown in Figure 9A. For the whole period of the compared cultures, the highest m_P_ values were identified for the culture system supported with xerogel A1 (Figure 9A). Compared to culture with xerogel A1, both systems of culture supported with gel B2 and reference culture were characterized by low values of m_P_. If comparing the m_P_ values reached in the gel-supported systems, the m_P_ values describing the culture with xerogel B2 were lower than those for the culture supported with xerogel A1. In the case of the control culture, the m_P_ value for the whole culture period was equal to 0 µg due to the level of deoxyshikonin being lower than the detection limit of the applied analytical method. Summarized production of deoxyshikonin, including bioproduct accumulated individually in hairy root biomass, xerogel, and culture medium taken from gel-supported systems, is presented in Figure 9B,C. In the case of culture supported with xerogel A1, up to the end of the 4th week of culture, almost the whole deoxyshikonin accumulated in the applied MTMS-based biomaterial (Figure 9B). However, in the following weeks, the amount of bioproduct accumulated in the biomass of transgenic roots increased. Finally, from the 5th week of culture, the amounts of deoxyshikonin accumulated in xerogel and biomass were equal. In the case of cultures supported with monolithic xerogel B2, the whole quantity of deoxyshikonin fully accumulated in the biomaterial (Figure 9C), as no bioproduct in biomass or culture medium was detected.

**Figure 10:**
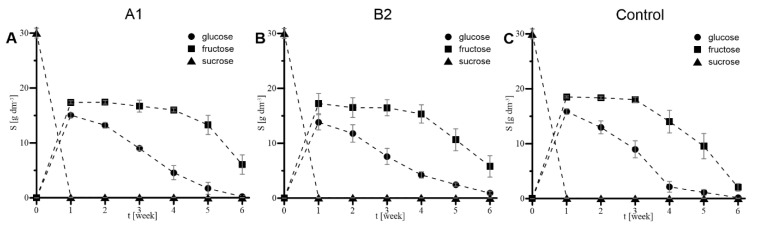
The concentration profiles of sucrose, glucose, and fructose characterizing the prolonged 42-day (i.e., 6-week) cultures supported with xerogels A1 (disintegrated mesoporous xerogel) (**A**) and B2 (PP-reinforced microporous xerogel) (**B**) and the control culture (**C**).

The concentration profiles of sucrose, glucose and fructose characterizing the prolonged 42-day (i.e., 6-week) cultures supported with xerogels A1 (A) and B2 (B) and the control culture (C) are presented in Figure 10. Regardless of the culture system, sucrose concentration dropped to 0 g L^−1^ during the first week due to the total hydrolysis of this disaccharide into glucose and fructose. Glucose assimilation occurs up to the 4th week of all cultures of *R. graeca* transgenic roots, which used this monosaccharide as a preferred carbon source. The concentration of fructose stayed almost unchanged over that time. A little difference was observed only in the 4th week of bioprocess by an apparent intensification of fructose assimilation of the control culture (Figure 10C) compared to relatively stable continued assimilation of glucose by biomass cultured in systems supported with MTMS-based materials, i.e., gels A1 (Figure 10A) and B2 (Figure 10B). Next, during weeks 5 and 6, the concentration of both glucose and fructose was intensively decreased in the culture media of the compared culture systems. Compared to gel-supported cultures, the control culture exhibited intensified glucose and fructose concentration decreases.

Values of µ, Y_P/X_, and Y_X/S_ obtained for the prolonged 42-day (i.e., 6 weeks) cultures of *R. graeca* hairy roots supported with xerogels A1 and B2, and the control culture, are presented in Table 2. The highest value of µ gave the culture with xerogel A1. For control culture and culture with xerogel B2, the µ values were equal, but they were almost 20% lower than the µ value characterizing culture supported with xerogel A1. The highest Y_P/X_ value was noted for the culture system supported with xerogel A1—the maximal value pointed out for this culture was over 12 times higher than the maximal value for the culture with xerogel B2. In the control culture case, the Y_P/X_ value was equal to 0 µg g_DW_^−1^ due to undetected deoxyshikonin in samples harvested from that culture. The values of Y_X/S_ were also calculated. The highest value of Y_X/S_ was observed for the culture supported with xerogel A1, and it was almost two times higher than those characterizing culture with xerogel B2. Both cultures gave higher Y_X/S_ values than the control culture.

## 3. Discussion

Porous MTMS-based materials showed exciting properties for the cultivation of transgenic roots and the secretion of secondary metabolites [21]. As a continuation of the research, it was proposed to screen for and further select the dry gel’s internal structure (morphology) and its form, i.e., PP/MTMS construct or disintegrated MTMS gel.

In the first stage of studies (i.e., the screening stage), a slight difference in FB values was observed for cultures supported by monolithic A2 and B2 MTMS-gel constructs. However, for cultures supported by MTMS-gel characterized by the same pores structure but varying in disintegrated form (i.e., A1 and B1 xerogels), the FB values were lower than for cultures containing A2 and B2 xerogel monolithic constructs (Figure 6). The differences between various forms of xerogels (i.e., monolithic and disintegrated forms) may result from mechanical protection and better access to oxygen and nutrients in cultures supported by MTMS-gel monolithic constructs. On the contrary, in the case of cultures containing monolithic forms of xerogel, the contact with the biomass hypothetically was limited to the material surface, which may result in less efficient sorption of secondary metabolites. On the other hand, the disintegrated gel did not support the roots, which floated in the medium only surrounded by particles of the xerogel. The advantage of the disintegrated xerogel is the larger interfacial surface contact area with the material, better metabolite secretion, and higher production of secondary metabolites, which is valid for all of the studied disintegrated forms of xerogels (Figure 7). The gel layer created by disintegrated xerogel can hypothetically limit oxygen and nutrient transport if particles of xerogel are densely packed.

The results confirm the hypothetical assumptions described above. Constructs provide better mechanical protection, higher growth but smaller root/xerogel contact surface, worse metabolite reception, and lower production (Figure 6 and Figure 7). The most pronounced production gave the xerogel with the smallest pores, A1, the desired effect of capillary pressure on metabolite reception [35,36,37]. The culture variant with C2 exhibited the lowest biomass growth, which suggests that the presence of xerogel is crucial. The non-woven PP alone or PP with a small amount of gel does not guarantee growth [21].

**Figure 11:**
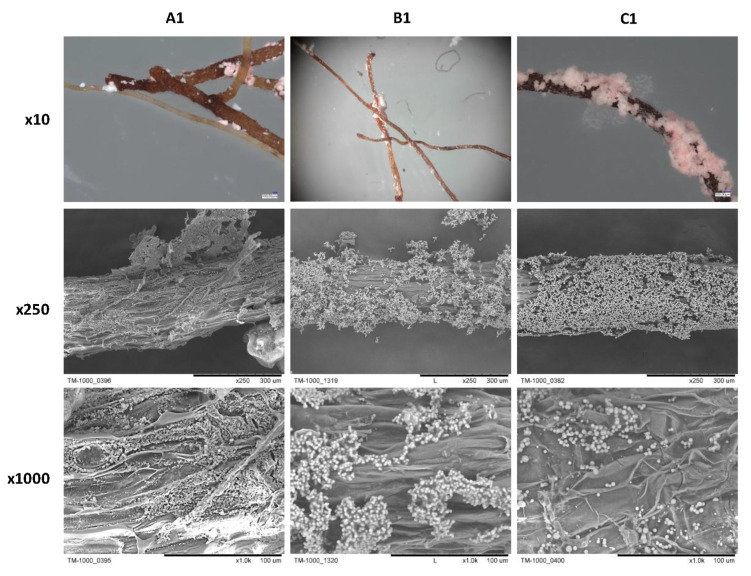
The optical microscope images (×10 magnification) and SEM micrographs (×250 and ×1000 magnifications) of hairy roots cultured in samples A1, B1, and C1.

The disintegrated gel’s morphology affects how it is arranged on the root surface, as shown in Figure 11. The gel of the A1 sample enters the unevenness on the root surface, while B1 and C1 form a loosely adjacent layer. The xerogel deposition differences affect the transport of the metabolite from the root to the sorbent. Hence, the highest production was observed for sample A1.

**Figure 12:**
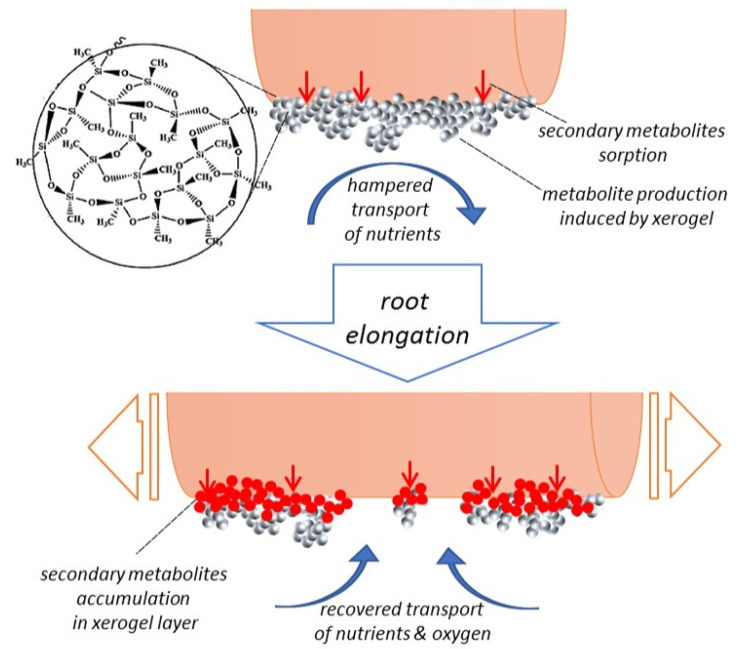
Schematic diagram showing the hypothetical effects of xerogel on the growth and production of metabolites in transgenic roots at different culture stages.

As described in Section 2.3, the highest increase in biomass was recorded for the A1 sample (after 6 weeks), suggesting that absorbing a toxic metabolite may be more important than mechanical protection in the case of prolonged cultures (i.e., 6 weeks). It is also possible that the presence of a metabolite close to the root surface (i.e., molecules of metabolite absorbed in gel) leads to increased stress and, therefore, boosts the production of naphthoquinones. Biomass growth and metabolite production accelerated between the 4th and 6th week. Initially, the gel structure covers the surface of the root well, and the availability of oxygen/medium constituents may be limited. During cultivation, the roots may be partially exposed after exceeding a certain root growth, and the availability of oxygen and medium components increases, accelerating the growth (Figure 12). Another explanation would be the importance of an in situ removal of secondary metabolic products, thus limiting their growth inhibitory effect. Further experiments are required to designate the occurring phenomena in detail.

The suitable way for discussing the effectivity of the culture systems supported with gels A1 and B2 in relation to the control culture is to directly compare the results presented in Figure 8 (i.e., profiles of FB), Figure 9 (i.e., a profile of deoxyshikonin), and Figure 10 (i.e., profiles of sugars). Generally, after comparison of the FB values with changes in carbohydrate concentrations, a decrease in the concentration of carbohydrates is visible with an increase in the FB value, regardless of the culture system. The observed intensification of the transgenic root growth between the 4th and 5th week of cultures supported by xerogel A1 (Figure 8A) may be related to the beginning of fructose consumption by the biomass maintained in this gel-supported system. However, for the culture system without any MTMS-based material (i.e., the control culture), a decrease in hairy root growth rate simultaneous to increased fructose consumption between the 5th and 6th week was observed (Figure 10B). In summary, it may be hypothesized that intensive biomass proliferation of hairy roots observed in the culture variant supported with disintegrated mesoporous xerogel A1 (Figure 8A) results from more efficient assimilation of glucose soluble in the culture medium (Figure 10A). At the same time, A1 mesoporous xerogel, which covers the roots to a greater extent, acts as an effective in situ extrahent and, by limitation of biosynthesis feedback inhibition, leads to its enhanced deoxyshikonin production.

The results proved the positive selective impact of the presence of the MTMS-based gel on the growth of *Rindera graeca* biomass and the intensification of the production of secondary metabolites. The results of the metabolites sorption emphasize the bifunctionality of the proposed MTMS-gel-based material. Results presented in Figure 9 show no product detection in the culture liquid. For B2, sample products were present in the construct only, while for A1—in the construct and roots. For sample A1, it was impossible to accurately separate the gel from the surface of the grown root so that the measured production may come from unremoved material saturated with metabolites. On the one hand, this emphasizes the need to refine the methodology of disintegrated material separation and, on the other hand, the compatibility of the proposed material with investigated biomass [38,39].

## 4. Materials and Methods

### 4.1. MTMS-Based Materials: Synthesis and Characterization

All xerogels were synthesized according to the two-step acid–base sol–gel methodology initially proposed by Bhagat and Rao [40] and improved by Nowak [32,41]. As an organosilica precursor, trimethoxymethylsilane MTMS (Sigma-Aldrich, Poznań, Poland) was used. As a solvent, methanol (Stanlab, Lublin, Poland) was applied. Oxalic acid (Sigma-Aldrich, Poznań, Poland) and ammonia water (Eurochem BGD, Tarnów, Poland) were applied as catalysts. The detailed proportions of precursor, solvent, and catalysts used to synthesize samples A-C of MTMS-based xerogel are shown in Table 3.

Two types of MTMS-based xerogel samples were applied in the experiments: disintegrated (by shredding) pieces of pure xerogels (marked as A1, B1, and C1) and monolithic xerogels reinforced with polypropylene (PP) non-woven fibrous mats (marked as A2, B2, and C2).

In the case of A1–C1 samples, xerogels were independently prepared in a sterile disposable sealed 100 mL jar-like container. First, MTMS was mixed with methanol and 0.01 M oxalic acid, and the mixture was vigorously stirred for 60 min to initiate the hydrolysis reaction. Then, 1 M ammonia solution was added to the mixture to start the gelation process. Condensation took place at room temperature. Next, the wet xerogels were transferred into a larger container with pure methanol for flushing samples. The solvent exchange was tripled to remove residual water, unreacted precursor, and catalysts. Samples were slowly dried at ambient pressure at 50 °C for four days (in a container with a loosened lid) and then at 100 °C for several hours (in a container without a lid) to evaporate any residual liquids from the xerogel pores. The samples of dried MTMS-based xerogels were characterized and then disintegrated in mortar to obtain the standard shredded xerogels marked as A1, B1, and C1, for direct application in hairy root cultures.

In the case of A2–C2 samples, the base material of non-woven PP-based fibrous mats was manufactured using melt-blown technology [42]. As a result, 50-layer filter mats (2.86 mm thick) were trimmed into circles with an effective diameter of 15 cm. The mean fiber diameter was determined from the scanning electron microscope (SEM) images as equal to 6.41 ± 4.18 μm. Finished PP-based mats were individually placed in disposable sealed 100 mL jar-like containers and soaked in 2-propanol to allow for better access by reagents during gelation. Then, the condensing solution (150 mL in each case) was poured onto the PP-based reinforcement and left for gelation. After a triple rinse in methanol and further two-step drying, the dried PP-reinforced MTMS-based constructs were cut into circles 4 cm in diameter. After detailed characterization, they were applied in hairy root cultures as samples marked A2, B2, and C2.

All dried samples of pure xerogels and PP-reinforced xerogel-based constructs were measured and weighed to designate their volume shrinkage, porosity (ε), and packing density (α), according to the following equations:

(1) $$\varepsilon =1-(m_{\mathrm{ag}\;}\times \;V_{\mathrm{ag}}{}^{-1}\;\times \;\rho_{\mathrm{sk}}^{\mathrm{ag}}{}^{-1})\;[\%],$$

(2) $$\alpha =((m_{\mathrm{PP}}\;\times \;\rho_{\mathrm{sk}}^{\mathrm{PP}}{}^{-1})+(m_{\mathrm{ag}}^{\mathrm{deposited}}\;\times \;\rho_{\mathrm{sk}}^{\mathrm{ag}}{}^{-1}))\;\times \;V^{-1}\;[g\;{\mathrm{mL}}^{-1}],$$

where m_ag_ and V_ag_ are xerogel sample mass and volume, respectively, m_PP_—mass of PP-based mat, $\rho_{\mathrm{sk}}^{\mathrm{PP}}\;$—PP skeletal density, $m_{\mathrm{ag}}^{\mathrm{deposited}}$—mass of deposited xerogel, $\rho_{\mathrm{sk}}^{\mathrm{ag}}\;$—xerogel skeletal density, V—volume of PP-reinforced MTMS-based xerogel construct.

The values of skeletal density of PP-based mat and MTMS-based xerogel were measured on a helium pycnometer (Humi-Pyc Model 2, InstruQuest Inc., Boca Raton, FL, USA), equaling 0.906 and 1.205 g mL^−1^, respectively.

The morphology of all tested materials was characterized via scanning electron microscope (SEM, Hitachi TM1000, Chiyoda, Tokyo, Japan). For the best resolution of SEM imaging, samples were firstly sputtered with a conductive chromium and gold nanolayer.

Five samples with the known weight of each piece were placed in a tested liquid (olive oil or rapeseed oil) for 24 h to estimate the tested samples’ adsorption capacity (AC). The values of AC were calculated as a mass of liquid per gram of material, as follows:

AC = (m_wet_ − m_dry_) × m_dry_^−1^ [g_oil_ g_material_^−1^], (3)

where m_wet_ is xerogel mass after adsorption of tested liquid, and m_dry_ is xerogel mass before liquid adsorption.

### 4.2. Biomass

In this study, *Rindera graeca* hairy roots were originally introduced to the in vitro conditions and were established as a stable culture by Katarzyna Sykłowska-Baranek et al. [43]. Before the developing culture systems supported with samples of xerogels, the inoculum of *R. graeca* hairy roots was maintained in 250 mL Erlenmeyer flasks filled with 50 mL of hormone-free DCR medium (PhytoTech Labs, Inc., Lenexa, KS, USA) [14]. The flasks were agitated on an oscillatory shaker ISS-7100 (Lab Companion, Billerica, MA, USA) at 105 rpm and 24 °C in darkness for 28 days. Proliferating hairy roots were passaged into new flasks containing fresh culture medium every four weeks, i.e., 28 days.

### 4.3. Xerogel-Supported Cultures of Hairy Roots

Experimental xerogel-supported cultures of *R. graeca* hairy roots were divided into two stages. In the first stage of the research, 28-day (i.e., 4-week) screening cultures investigating the physiological effects of hairy roots after supplementing the cultures with various xerogel forms were performed. In the second stage of the research, 42-day (i.e., 6-week) prolonged cultures investigating the detailed impacts of xerogel forms selected in the screening studies were performed. Regardless of the research stage, all culture systems were developed in the 250 mL Erlenmeyer flasks containing the 50 mL fresh DCR culture medium. Flasks were mounted on the oscillatory shaker and incubated in darkness at a temperature of 24 °C under continuous oscillatory shaking at 105 rpm.

All tested materials were sterilized by 25 min of autoclaving at 121 °C before introducing them into culture systems. All culture systems were developed sterile under laminar flow conditions, where samples of materials were placed inside the 250 mL Erlenmeyer flasks containing the 50 mL of fresh DCR culture medium as follows: 1.0 g of disintegrated xerogel (i.e., samples A1–C1) or one PP/MTMS construct (i.e., samples A2–C2) per flask. Next, 1.0 g of *R. graeca* hairy roots were used as inoculum and inserted sterile into each culture system: added directly into the culture medium containing elements of disintegrated xerogel (i.e., samples A1, B1, C1) or placed precisely on the surface of PP/MTMS constructs (i.e., samples A2, B2, C2). Visual analysis of root biomass and xerogel interactions after the cultures was performed with Keyence VHX-7000 digital microscope (Mechelen, Belgium) assistance.

### 4.4. Phytochemical Analysis

Dried extracts obtained from leached biomass, xerogel-based materials, and extracted culture medium were independently redissolved in methanol (HPLC grade) before analytical procedures. Redissolved samples were analyzed by reversed-phase HPLC (RP-HPLC) chromatographic technique using the DIONEX 3000 HPLC system (by Dionex, a brand of Thermo Scientific, Waltham, MA, USA) supported with EC Nucleosil 120-7 ODS column (250 × 4.6 mm, 7 μm particles, 120 Å pores, Macherey-Nagel, Allentown, PA, USA), and equipped with UV–Vis diode-array detector (UVD 340S) and automated sample injector (ASI-100). For chromatographic separation performed under gradient elution, a mixture of acetonitrile (60–80%)/0.04 M orthophosphoric acid (40–20%) at a flow rate of 1.5 mL min^−1^ was applied as a continuous phase. Eluent absorbance was monitored at 215, 237, 350, and 436 nm. The concentration of deoxyshikonin in samples was quantitatively estimated by analysis of specified peaks at 215 nm wavelength on chromatograms according to the standard external method. Deoxyshikonin standard of confirmed identity was used for qualitative peak identification and calibration curve preparation for quantitative analysis.

### 4.5. Carbohydrates Concentration Analysis

The concentrations of carbohydrates in the second stage of the research, i.e., 6-week prolonged cultures, were measured by HPLC chromatographic technique using the Varian 635 CL System (Varian Inc., Palo Alto, CA, USA) supported with a 60 °C thermostat-controlled Rezex RSO-Oligosaccharide Ag+ 4% column (Phenomenex Inc., Torrance, CA, USA) and equipped with the Smartline 2300 refractive index (RI) detector (Knauer, Berlin, Germany). For chromatographic separation, distilled water at a flow rate of 0.4 mL min^−1^ was used. The concentration of carbohydrates, i.e., glucose, fructose, and sucrose, was determined based on previously prepared standard curves.

### 4.6. Mathematical Methods

The proliferation of the *R. graeca* transgenic roots was quantitatively identified by the values of the fresh biomass increase at n-day of the culture (FB_n-day_) and the dry biomass increase at n-day of the culture (DB_n-day_), which were determined based on the following equations:

(4) $${\mathrm{FB}}_{n-\mathrm{day}}=m_{n-\mathrm{day}}^{\mathrm{FB}}\;\times \;m_{0}^{\mathrm{FB}-1}\;[-],$$

where $m_{n-\mathrm{day}}^{\mathrm{FB}}$ is the fresh biomass weight for the sample harvested at n-day of culture, and $m_{0}^{\mathrm{FB}}\;$is the fresh biomass weight of inoculum;

(5) $${\mathrm{DB}}_{n-\mathrm{day}}=m_{n-\mathrm{day}}^{\mathrm{DB}}\;\times \;m_{0}^{\mathrm{DB}}{}^{-1}\;[-],$$

where $m_{n-\mathrm{day}}^{\mathrm{DB}}\;$is the dry biomass weight for the sample harvested at n-day of culture, and $m_{0}^{\mathrm{DB}}\;$denotes the dry biomass weight of the inoculum.

**Figure 13:**
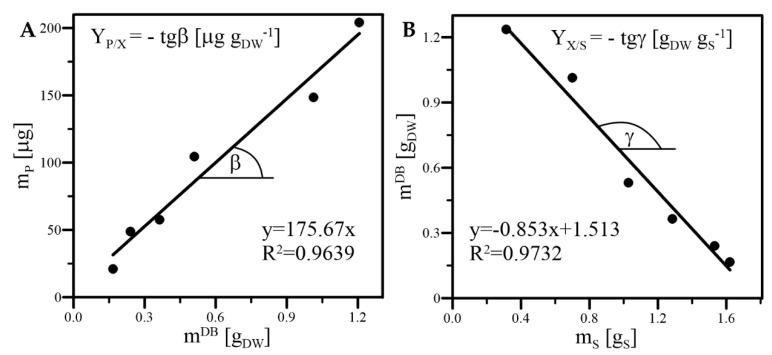
Exemplary determination of Y_P/X_ (**A**) and Y_X/S_ (**B**) values by the graphical methods based on quantitative data collected for the culture of *R. graeca* hairy roots supported with A1 form of MTMS-based xerogel.

The yield of deoxyshikonin per dry biomass weight (Y_P/X_) is defined as the total mass of deoxyshikonin (i.e., the product) available in an individual culture system (m_P_) produced by 1.0 g of dry biomass of hairy roots. Values of Y_P/X_ were determined by the graphical method (Figure 13A) according to the following equation:

(6) $$Y_{P/X}{\;}_{\;}=m_{P}\;\times \;{\left(m_{n-day}^{DB}-m_{0}^{DB}\right)}^{-1}=\mathrm{tg}\beta \;[µg\;{g_{\mathrm{DW}}}^{-1}],$$

The yield of biomass obtained from assimilated carbohydrates (Y_X/S_), describing the increase in the dry weight of hairy roots resulting from the assimilation of 1 g of carbon substrate, was determined by the graphical method (Figure 13B) according to the following equation:

(7) $$Y_{X/S}=(m_{n-\mathrm{day}}^{\mathrm{DB}}-{\;m}_{0}^{\mathrm{DB}})\;\times \;{\left(m_{S0}-m_{\mathrm{Sn}-\mathrm{day}}\right)}^{-1}=-\mathrm{tg}\gamma \;[g_{\mathrm{DW}}\;{g_{S}}^{-1}],$$

where $m_{\mathrm{Sn}-\mathrm{day}}\;$is the total carbohydrates mass for the sample harvested at n-day of culture, and $m_{S0}\;$stands for the total carbohydrate mass at starting day of culture. The $m_{\mathrm{Sn}-\mathrm{day}}$ is a sum of all carbohydrates, i.e., glucose, fructose, and sucrose, concentration (S) at n-day of culture multiplied by the volume of culture medium (i.e., 50 mL).

To quantitatively describe the growth rate of *R. graeca* hairy roots in studied systems, the values of specific growth rate (µ) were determined according to the following equation:

(8) $$µ=[\ln\left(m_{n-\mathrm{day}}^{\mathrm{DB}}\right)-\ln\left(m_{0}^{\mathrm{DB}}\right)]\;\times \;{\left(\Delta t\right)}^{-1}\;[h^{-1}],$$

where Δt is the time of culture expressed in hours.

### 4.7. Statistical Analysis

The statistical difference for the mean values of root biomass growth and deoxyshikonin productivity were tested with a one-way analysis of variance (ANOVA). The Shapiro–Wilk test and Bartlett’s test were applied for normal distribution and variance homogeneity, respectively. For all experiments, *p* ≤ 0.05 was considered significant.

## 5. Conclusions

Plant secondary metabolites are significant products for many industries interested in efficient manufacturing. However, in many cases, the complex chemical structures, low natural productivity, or ecological limitations make it unfeasible for commercial application. Considering the great value of these compounds, the search for more efficient methods of industrial production is highly justified. The presented study demonstrates the successful example of novel silica xerogel materials application for in situ extraction of naphthoquinone compound, deoxyshikonin, in in vitro culture systems of *Rindera graeca* hairy roots. The investigation covered the MTMS-based xerogel morphology effects on the *R. graeca* transgenic roots growth kinetic and deoxyshikonin production and their selectivity. The monolithic PP fiber-reinforced gels protected the fragile biomass of hairy roots against hydrodynamic stress and promoted plant biomass growth. The disintegrated gels significantly intensify secondary metabolite production. The mesoporous disintegrated MTMS xerogel gave the highest values of the deoxyshikonin output, characterized by the smallest pore size and, thus, the highest capillary pressure of studied xerogels. In addition, in the case of all disintegrated xerogels, the deposition of particles suspended in the medium occurs on the root tissue surface, presumably improving the sorption of secondary metabolites from biomass. On the other hand, the homogenous layer of the deposited xerogel might negatively affect the transport of nutrients and oxygen from the medium to the root, which correlates with the lower values of biomass growth during the initial period of culture. The interaction between xerogel and hairy root biomass requires further investigation.

## 6. Patents

PL Patent P-437075, 22.02.2021. Application of methyltrimetoxysilane-based organosilica aerogels for proliferation and immobilization of plant biomass cultured in vitro and application of methyltrimetoxysilane-based aerogels for intensification of plant metabolite production and intensification of in situ extraction of secondary metabolites in in vitro cultures.

## Figures and Tables

**Table 1:** Characterization of PP non-woven mat and PP-reinforced MTMS-based constructs.

Material Sample	Volume Shrinkage (%)	Packing Density (g mL^−1^)	MTMS-Gel/PP Fibers Weight Ratio (g g^−1^)
PP	-	8.11 (±0.87)	-
A2	58.00 (±0.12)	86.49 (±2.40)	5.26 (±0.36)
B2	28.42 (±0.09)	43.47 (±3.11)	4.35 (±0.31)
C2	1.32 (±0.08)	14.45 (±5.21)	1.29 (±0.18)

**Table 2:** Values of µ, Y_P/X_, and Y_X/S_ obtained for the prolonged 42-day (i.e., 6 weeks) cultures of *R. graeca* hairy roots supported with xerogels A1 and B2 and the control culture.

Kinetic Parameter	Xerogel A1	Xerogel B2	Control
µ (h^−1^)	0.00205 (±0.00045)	0.00172 (±0.00025)	0.00172 (±0.00034)
Y_P/X_ (µg g_DW_^−1^)	175.67 (± 5.71)	14.48 (± 1.08)	0.00 (±0.00)
Y_X/S_ (g_DW_ g_S_^−1^)	0.853 (±0.023)	0.463 (±0.017)	0.328 (±0.011)

**Table 3:** Molar and volumetric ratios applied in xerogel synthesis.

MTMS-Based Xerogel Sample	MTMS:MeOH:C_2_H_2_O_4_^(1)^:NH_4_OH ^(2)^ Volumetric Ratio (mL)	MTMS:MeOH:H_2_O:C_2_H_2_O_4_:NH_4_OH Molar Ratio (moles)
A	1.0:2.0:0.5:0.5	1.00:7.05:11.56:1.00∙10^−3^:1.14
B	1.0:2.0:0.8:0.8	1.00:7.05:7.22:1.00∙10^−3^:0.71
C	1.0:10.0:4.0:4.0	1.00:35.26:57.78:6.00∙10^−3^:5.71

^(1)^ 0.01 M C_2_H_2_O_4_ ^(2)^ 1 M NH_4_OH.

## Data Availability

The data presented in this study are available on request from the corresponding author.
